# Editorial: Mind the Heart – Psychosocial Risk Factors and Cognitive Functioning in Cardiovascular Disease

**DOI:** 10.3389/fpsyg.2021.670235

**Published:** 2021-04-30

**Authors:** Edward Callus, Giada Pietrabissa, Noa Vilchinsky

**Affiliations:** ^1^Clinical Psychology Service, IRCCS Policlinico San Donato, Milan, Italy; ^2^Department of Biomedical Sciences for Health, University of Milan, Milan, Italy; ^3^Psychology Research Laboratory, Istituto Auxologico Italiano IRCCS, Milan, Italy; ^4^Department of Psychology, Catholic University of Milan, Milan, Italy; ^5^Department of Psychology, Bar-Ilan University, Ramat Gan, Israel

**Keywords:** cardiovascular disease, psychosocial factors, cognitive deficit, psychotherapy, rehabilitation

Cardiovascular diseases (CVDs) are the leading cause of disability and premature death worldwide, and they contribute substantially to rising health care costs. Ameliorating cardiac risk factors is the most effective way of minimizing CVDs' negative physical and psychological ramifications World Health Organization (WHO, 2021). By targeting those people who are most predisposed to develop cardiac illness or whose risk factors prevent them from managing their illness as recommended, we might be able to minimize the vast global burden of this illness. The current topic for *Frontiers in Psychology* presents an effort to gain an integrated view of the role played by psychosocial and cognitive factors in the context of CVD.

The main variables analyzed in the current collection were anxiety, depression, and stress (Grech et al.; Murphy et al.; Callus et al.; Eisenberg et al.; Ely et al.; Heenan et al.). The main tools presented for measuring these construct were the DASS-21 (Grech et al.; Eisenberg et al.), the HADS (Murphy et al.; Heenan et al.), the PHQ-9 (Grech et al.; Callus et al.; Ely et al.), and the GAD-7 (Grech et al.; Callus et al.; Ely et al.). Less observed psychosocial variables were those capturing well-being, as measured by the PGWBI (Pietrabissa et al.) and the SF-36 (Heenan et al.), and those focusing on behaviors as nutritional behaviors, measured by the healthy heart score (Eisenberg et al.). In the rare occasions where patients' cognitive abilities were measured, the researchers applied the NAB (Neuropsychological Assessment Battery) (Byron-Alhassan et al.) or the Mini-Cog, a test used for memory evaluation (Ely et al.).

These psychosocial risk factors were found to be associated with CVD either directly or indirectly via unhealthy behaviors which are known to contribute to cardiac morbidity and mortality. For example, Murphy et al., detected anxiety and depression among 15–43% of their sample of 911 patients coping with acute myocardial infarction (AMI). Heenan et al. detected that cardiac patients' anxious attachment orientation has been associated with anxiety, depression, poorer physical and mental quality of life, and even fasting blood glucose, HbA1c, via greater levels of traumatic stress. In addition, psychological distress was associated with obesity among heart failure patients (Ely et al.), and psychological well-being was associated with exercise capacity- a well-established predictor of cardiovascular health (Pietrabissa et al.). Moderate (but not high) levels of anxiety were associated with consumption of sweetened drinks and white bread among a sample of women at cardiovascular risk (Eisenberg et al.). Somewhat surprisingly, a systematic review of case-control studies conducted by Galli et al. did not detect any consistent psychosocial risk factors associated with the emergence of Takotsubo syndrome.

Risk factors were explored in different CVD diagnoses, and different ages. Each population presented a unique picture of psychosocial and cognitive needs and difficulties. For example, Tye et al. revealed five themes of challenges adolescents with congenital heart defect face with: (1) emotional/psychological issues; (2) the progress of the illness; (3) relationship issues; (4) future preparation; and (5) school and community. Journiac et al. focused on young adults with congenital heart disease and showed that this age group copes with additional challenges as social isolation, identity changes and difficulties at work. On the other end of the spectrum, elderly cardiac patients cope with the additional burden of neuropsychological difficulties (Granata et al.). Indeed, Byron-Alhassan et al. detected regional atrophy in the brains of patient who underwent cardiac arrest and myocardial infarction, compared to a healthy control group. Overall, the current studies provide some useful insights for future research. First, in order to provide an adequate assessment of patient's emotional state, it is important to apply longitudinal designs as suggested in many of the current reports e.g., (Grech et al.). It is also recommended to prefer psychological interviewing over self-report questionnaires, due to the latter being much more susceptible to social desirability. Another advantage of applying an interview is the opportunity for the clinician who collects patient's information to also offer him/her with much needed support (Callus et al.; Granata et al.). There is also a need to launch broader, multicenter-based studies (national or international), integrating the biopsychosocial and the multidimensional approaches, and involving a more heterogeneous sample (Heenan et al.).

In the studies examined, attention was mainly paid to certain aspects of mental health, such as anxiety and depression, but future research should also consider aspects related to other diagnoses as post-traumatic stress disorder (Journiac et al.), as well as to patients' self-management acts as eating behavior (Eisenberg et al.) and physical exercise (Pietrabissa et al.). Finally, given the crucial role of early treatment in determining a better quality of life and less use of health services (Murphy et al.), it may be useful to focus on early identification of risk factors frequently present in CVD patients, so as to intervene in a timely manner.

To sum up, the accumulated data attest to the high prevalence of emotional distress among cardiac patients, which emerges as a consequence of cardiac illness and also plays a major role as risk factor for low adherence to health-promoting behaviors. Overall, cardiac patients seem to be caught in a vicious cycle in which negative psychological factors preceding the cardiac event or induced by it are associated with the emergence of additional risk factors, which down the road contribute to recurrence, and worse prognosis. Mapping and understanding patients' cognitive and emotional factors may be a valuable step in developing tailored primary and secondary cardiac prevention interventions. Overall, the assessment of psychological risk factors must be integrated into patients' health care routine during hospitalization, before discharge, and during convalescence, using designated tools and protocols.

## Author Contributions

EC produced the first draft. GP and NV reviewed the article. All authors contributed to the article and approved the submitted version.

## Conflict of Interest

The authors declare that the research was conducted in the absence of any commercial or financial relationships that could be construed as a potential conflict of interest.
